# Supplementary material for: Identification of photoperiod-induced specific miRNAs in the adrenal glands of Sunite sheep (Ovis aries)
Source: Front Vet Sci. 2022 Jul 22;9:888207. doi: 10.3389/fvets.2022.888207 (PMC9354845; doi:10.3389/fvets.2022.888207)
Supplement: Supplementary file 1 [file Data_Sheet_1.ZIP › Supplementary file/miRDeep2 file.zip!/miRDeep2 file/Know_Structure/SPLP42A3/oar-mir-362.pdf]

miRBase precursor : oar-mir-362
Total read count : 522
oar-miR-362 read count : 517
remaining reads : 5

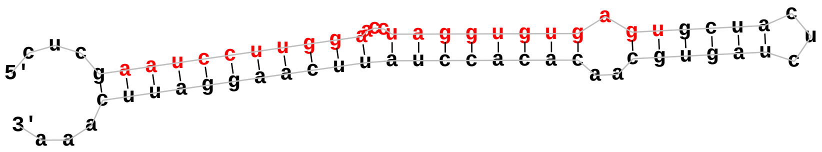

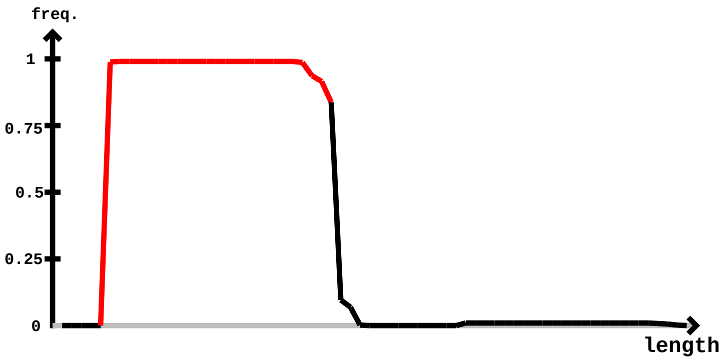


```
      oar-miR-362
5'- cucgaauccuuggaaccuaggugugagugcuacucuagugcaacacaccuauucaaggauucaaa   -3'    exp
    ...(((((((((((...((((((((.((((((...))))))..)))))))))))))))))))...    reads      mm         sample
    ....aauccuuggaaccuaggugu.........................................    2          0          seq
    ....aauccuuggaaccuaggugug........................................    26         0          seq
    ....aauccuuggaaccuagguguga.......................................    11         0          seq
    ....aauccuuggaaccuaggugugag......................................    41         0          seq
    ....aauccuuggaaccuaggugugagu.....................................    386        0          seq
    ....aauccuuggaaccuaggugugagug....................................    14         0          seq
    ....aauccuuggaaccuaggugugagugc...................................    35         0          seq
    ....aauccuuggaaccuaggugugagugcu..................................    1          0          seq
    .....auccuuggaaccuaggugugagu.....................................    1          0          seq
    .........................................aacacaccuauucaaggauu....    1          0          seq
    .........................................aacacaccuauucaaggauuc...    1          0          seq
    .........................................aacacaccuauucaaggauuca..    2          0          seq
    .........................................aacacaccuauucaaggauucaa.    1          0          seq
```
